# Clinical, laboratory and ultrasonographic findings in 38 calves with type-4 abomasal ulcer

**DOI:** 10.1186/s13028-021-00601-2

**Published:** 2021-09-25

**Authors:** Ueli Braun, Christina Widmer, Karl Nuss, Monika Hilbe, Christian Gerspach

**Affiliations:** 1 Department of Farm Animals, Vetsuisse Faculty, University of Zurich, Zurich, Switzerland; 2 Institute of Veterinary Pathology, Vetsuisse Faculty, University of Zurich, Zurich, Switzerland

**Keywords:** Abomasum, Calf, Peritonitis, Type-4 ulcer

## Abstract

**Background:**

Type-4 abomasal ulcers (U4) are perforated ulcers causing diffuse peritonitis. This retrospective study describes the clinical, laboratory and ultrasonographic findings in 38 calves with U4. The medical records of 38 calves aged three days to 20 weeks with U4 were scrutinised.

**Results:**

The most common clinical findings were poor general health (95%), reduced skin elasticity (95%), rumen atony (91%), abdominal guarding (76%) and positive percussion auscultation and/or swinging auscultation on the right side of the abdomen (75%). The most frequent laboratory findings were increased numbers of segmented neutrophils (87%), eosinopenia (87%), acidosis (84%), azotaemia (79%) and hyponatraemia (79%). The most frequent abdominal ultrasonographic findings were intestinal atony (68%), fluid (67%) and fibrin deposits (58%) in the abdomen. Thirty-five calves were euthanased and three calves died spontaneously. All calves underwent pathological examination. Diffuse peritonitis caused by a transmural abomasal ulcer was the principal diagnosis in all calves.

**Conclusions:**

Perforated abomasal ulcers cause severe illness, and a thorough clinical examination combined with ultrasonographic abdominal examination should lead to a tentative diagnosis.

## Background

Postmortem studies have shown that perforated abomasal ulcers are an important cause of death in veal calves, and in one report accounted for 22% of all losses [1]. However, fatal ulceration is merely one part of the problem but has serious consequences [2, 3] because the prevalence of non-fatal, non-perforated ulcers is high and ranges from 16.7% (fundic region [2]) to 36% (fundic or pyloric region) [4], 59.3% (fundic or pyloric region) [5], 74.1% (pyloric region) [6] and 80% (fundic or pyloric region) [7]. Abomasal ulcerative lesions in veal calves are classified as erosions, ulcers and scars [3, 8] but in principle could also be divided into types 1 (U1) to 5 (U5) analogous to what was recently described in detail for U1 [9], U2 [10], U3 [11], U4 [12] and U5 [13] in cows. Abomasal ulcers of veal calves are divided into pyloric and fundic ulcers [3, 14]. A detailed overview of risk factors for abomasal ulcers in calves, including nutritional aspects, stress, comorbidity and medical treatment, was recently published [3].

A study of 118 calves with abomasal ulceration identified an increased prevalence in calves aged 5–12 weeks [15]. At that time, the calves were weaned and had started to eat larger amounts of solid feed. It was therefore surmised that the incompletely developed forestomachs allowed the transport of poorly digested feed into the abomasum causing damage to the mucosa. The notion that abomasal overloading associated with feeding large amounts of milk favours the development of abomasal ulcers [16, 17] is not supported by scientific evidence [3]. An experimental study [18] and a risk assessment study [6] did not support the notion that overloading of the abomasum by high milk volumes delivered in a small number of meals per day was responsible for abomasal ulcers. The prevalence of abomasal lesions was also increased in calves on a high nutritional plane and with a high carcass weight [19, 20]. Low feeding frequency (twice daily versus ad libitum) [21] and feeding of straw [22, 23] or corn silage [24, 25] are other risk factors for abomasal ulceration. Veal calves raised in production programs that are in compliance with increased animal welfare requirements had significantly fewer ulcers in the fundic part of the abomasum than calves raised conventionally under conditions with minimal animal welfare standards [20]. Use of nonsteroidal anti-inflammatory drugs (NSAIDs) has also been associated with an increased prevalence of abomasal ulcers in calves [26, 27]. The role of microbials such as *Clostridium perfringens* and *Campylobacter jejuni* in the pathogenesis of abomasal ulcers is controversial and shall not be discussed here.

This study is limited to calves affected by type-4 abomasal ulcers (U4), which are perforated ulcers causing diffuse peritonitis. The main clinical findings of 299 calves [15] and the laboratory and pathological findings of 81 [25] and 80 calves [28] with perforated ulcers have been described, and other older publications on these topics reviewed [29]. The diagnosis of perforated abomasal ulcer in calves is not always straightforward and even though the clinical signs are severe, they are often misinterpreted. Therefore, the goal of this study was to describe the clinical, laboratory and ultrasonographic findings in 38 calves with U4 to facilitate rapid diagnosis and humane euthanasia in the field.

## Methods

### Calves

This was a retrospective study of 38 calves with a main diagnosis of U4. The calves had been admitted to the Veterinary Teaching Hospital, University of Zurich, from January 1, 1992 to December 31, 2017. The final diagnosis of U4 was based on the results of postmortem examination. The methods and the results were described in detail in a Master’s thesis [30]. The calves ranged in age from 3 days to 20 weeks (median  =  8 weeks). Breeds included Brown Swiss (n  =  23, 61%), Swiss Fleckvieh (n  =  4, 10%), Holsteins (n  =  3, 8%) and crossbred cattle (n  =  8, 21%). Twenty-three calves (61%) were replacement dairy heifers, 10 (26%) were veal calves and 5 (13%) were nursing beef calves. All calves underwent the same structured clinical and laboratory examination procedures. The ultrasonographic examination was less structured; intestinal motility was monitored in only 28 of 36 scanned calves.

### History

Thirty-six calves were not weaned and fed milk or milk replacer. Thirty-three calves had a complete feeding history with regard to other feeds; 27 of these were fed hay (n  =  25), grass and/or corn silage (n  =  9), mixed grain (n  =  8) or grass (n  =  3), whereas the remaining six calves did not receive additional feeds. Twenty-five calves received veterinary treatment before admission, which included antibiotics (n  =  14), NSAIDs (n  =  20) or corticosteroids (n  =  5). The duration of illness at the time of admission ranged from 6 to 21 days (median  =  1 day) but exceeded 4 days in only 3 calves.

### Inclusion criteria

Calves between 1 day and 6 months of age, in which U4 was diagnosed during laparotomy and/or pathological examination, were included in this study.

### Clinical examination

Clinical examination was done as described [31]. Rumen fluid was collected from 27 calves, and the physical characteristics including colour, odour and pH were determined. Voided urine from 20 calves was analysed using test strips (Combur9^®^, Roche, Basel).

### Haematological examination

The following blood samples were collected from the jugular veins of 37 calves: 5 ml of EDTA blood for haematological analysis, 5 ml of whole blood for serum biochemistry and 2 ml of whole blood mixed with 0.2 ml heparin for venous blood gas analysis. Haematological analysis included the determination of haematocrit and total leukocyte count using an automated blood analyser (CELL-Dyn 3500, Abbott Diagnostics Division, Baar, Switzerland). A white blood cell count differential was done in 23 calves. The concentrations of total protein, fibrinogen, serum urea, bilirubin, sodium, potassium, chloride, calcium, magnesium and inorganic phosphorus were determined at 37 °C using an automated analyser (Cobas-Integra-800-Analyser, Roche Diagnostics, Basel, Switzerland) and the manufacturer’s reagents (Roche Reagents) according to the International Federation of Clinical Chemistry and Laboratory Medicine (IFCC). Venous blood gas analysis was done using an automated analyser (RapidLab 248, Siemens Schweiz AG, Zurich, Switzerland).

### Ultrasonographic examination of the abdomen and abdominocentesis

Abdominal ultrasonography was done in 36 calves using a 5.0–7.5-MHz linear or convex transducer [32], and ultrasound-guided abdominocentesis was carried out in 15 calves.

### Euthanasia and postmortem examination

Thirty-five calves were euthanased using pentobarbital administered intravenously at 80 mg/kg body weight (Esconarkon^®^, Streuli Pharma AG, Uznach, Switzerland), 12 of these underwent exploratory laparotomy to obtain or confirm a diagnosis of U4. Three calves died spontaneously during or after the examination. All calves underwent pathological examination.

### Statistical analysis

All records were stored as PDF files, and the key findings and diagnoses were also stored in a FileMaker database. This allowed straightforward identification of calves with U4 from the more than 30,000 records. The clinical, laboratory and ultrasonographic findings of the calves were transferred to specifically designed protocol charts and analysed using SPSS Statistics 25.0 (IBM Corp. 2017, USA). First, the frequency distributions for all variables were calculated. The Shapiro–Wilk test was used to test data for normality; normal data are shown as means  ±  standard deviation, and non-normal data as median and 25th and 75th percentiles.

## Results

### Clinical findings

**Fig. 1:**
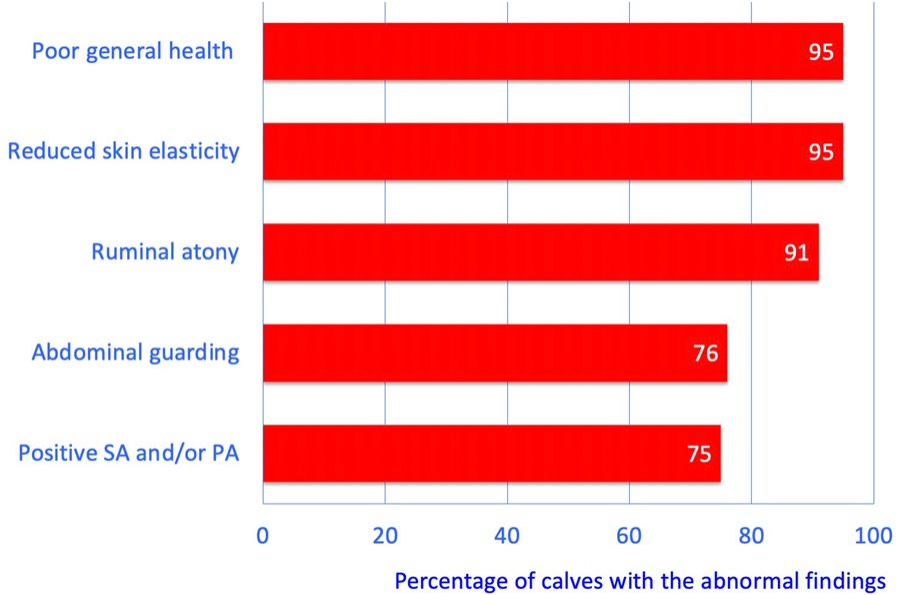
Abnormal clinical findings in 38 calves. Most common abnormal clinical findings in 38 calves with type-4 abomasal ulcer. PA/SA Percussion auscultation/swinging auscultation on the right side

The most common clinical findings in decreasing frequency were poor general health (95%), reduced skin elasticity (95%), rumen atony (91%), abdominal guarding (76%) and positive swinging auscultation, percussion auscultation or both on the right side of the abdomen (75%) (Fig. 1). The general condition was moderately (n  =  2, 5%) or severely affected (n  =  36, 95%) in all calves; 25 (65.8%) were listless, one calf (2.6%) was comatose and ten (26.3%) were recumbent. Four calves (10%) were thin, 14 (37%) had moderate and 20 (53%) good body condition. Nineteen (50%) calves had abdominal dilatation. The heart rate ranged from 68 to 180 beats/min (mean  ±  standard deviation: 124  ±  30 beats/min), the respiratory rate ranged from 20 to 102 breaths/min (median: 40 breaths/min) and the rectal temperature ranged from 35.8 to 40.1 °C (median: 39.2 °C) (Table 1). The heart rate was increased in 25 (66%), the respiratory rate in 13 (35%) and the rectal temperature in 8 (21%) calves. The rectal temperature was subnormal in 10 (26%) calves.

**Table 1:** Clinical findings in 38 calves with type-4 abomasal ulcer

Variable	Finding	n	%
General demeanour (n = 38)	Moderately disturbed	2	5
	Severely disturbed	36	95
Heart rate (n = 38; mean ± sd, 124 ± 30 bpm)	Normal (90–110)	9	24
	Decreased (68–89)	4	10
	Increased (111–180)	25	66
Respiratory rate (n = 38; median, 40 breaths per min)	Normal (30–45)	11	30
	Decreased (20–29)	13	35
	Increased (46–102)	13	35
Rectal temperature (n = 38; median, 39.2 °C)	Normal (38.5–39.5)	20	53
	Decreased (35.8–38.5)	10	26
	Increased (38.6–40.1)	8	21
Rumen (n = 35)	Normal motility	1	3
	Decreased motility	2	6
	Absent motility	32	91
	Rumen tympany	20	57
Foreign body tests^a^ (n = 23)	All negative	8	35
	At least one test positive^b^	15	65
Swinging and percussion auscultation on the right side (n = 36)	Both negative (normal)	9	25
	Only swinging auscultation positive	18	50
	Both tests positive	9	25
Swinging and percussion auscultation on the left side (n = 37)	Both negative (normal)	12	32
	Only swinging auscultation positive	12	32
	Only percussion auscultation positive	1	3
	Both tests positive	12	32
Intestinal motility (n = 37)	Normal	4	11
	Reduced	26	70
	Absent	7	19
Faeces (n = 38)	No faeces in the rectum	8	21
	Dark to black	6	16
	Soft to liquid	4	11
	Melaena or mucus or fibrin	13	34

^a^Foreign body tests: back grip, pole test, pain percussion

^b^Positive: at least 3 of 4 attempts elicited a grunt

Thirty-two of 35 (91%) calves had rumen atony (Table 1), and 20 (63%) calves had ruminal tympany. At least one foreign body test (back grip, pole test, pain percussion) was positive in 15 of 23 (65%) calves. Percussion and simultaneous auscultation and/or ballottement and simultaneous auscultation were positive on the right side in 27 (75%) and positive on the left side in 25 (66%) calves. Intestinal motility was reduced or absent in 33 (89%) calves.

The rectum was empty in 8 (21%) calves and contained dark or black faeces in 6 (16%) and loose or watery faeces in another 4 (11%). Blood, mucus or fibrin was found in the rectum of 13 (34%) calves.

Other abnormal findings were enophthalmus (n  =  22, 73%), prolonged capillary refill time (n  =  24, 71%), congested scleral vessels (n  =  25, 70%), dry and/or cold muzzle (n  =  21, 70%), decreased skin surface temperature (n  =  25, 66%) and pale oral mucosa (n  =  20, 53%).

Non-specific signs of pain including spontaneous grunting and bruxism were seen in 14 (37%) and 4 (11%) calves, respectively, and signs of parietal pain including abdominal guarding, arched back and tucked-up abdomen occurred in 29 (76%), 6 (16%) and 3 (8%) calves, respectively.

Urine pH was between 5 and 6 in 17 (85%) and between 7 and 8 in 3 (15%) calves. Five urine samples (25%) contained  ++  protein (approximately 100 mg protein/100 mL), and 6 (30%) were positive for erythrocytes or haemoglobin/myoglobin.

The ruminal fluid from 7 of 25 (28%) calves had a milky grey discolouration and a sour odour, and the pH was in the acidic range at 3.5–6.0 in 9 of 27 (33%) samples.

### Laboratory findings

**Fig. 2:**
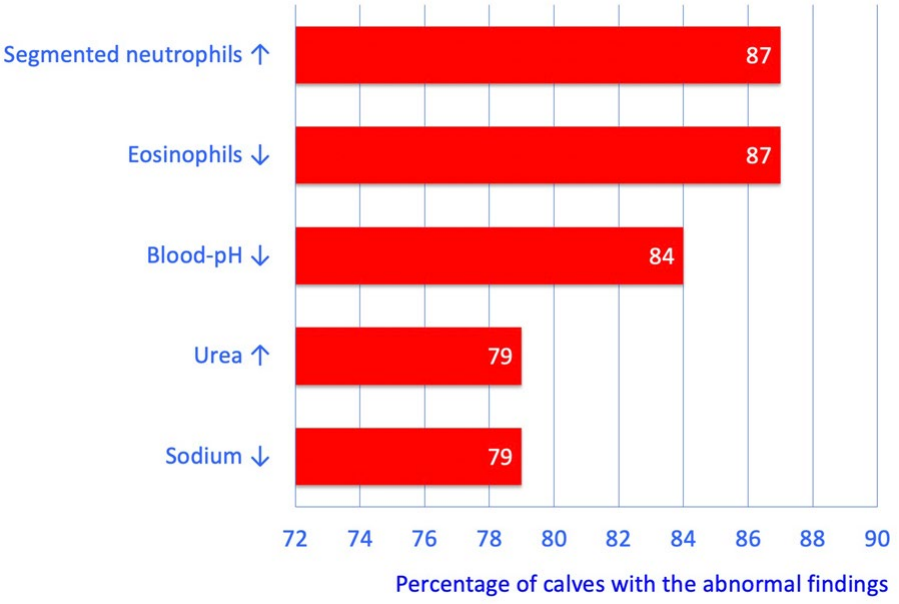
Abnormal blood variables in 38 calves. Most common abnormal laboratory findings in 38 calves with type-4 abomasal ulcer

The most common laboratory findings were increased numbers of segmented neutrophils (87%), eosinopenia (87%), acidosis (84%), azotaemia (79%) and hyponatraemia (79%) (Fig. 2). Haemoconcentration was seen in 22 (59%), hypoproteinaemia in 23 (62%) and hyperfibrinogenaemia in 10 (30%) calves (Table 2). The serum urea concentration was increased in 27 (79%) calves, and the most prevalent serum electrolyte imbalances were besides hyponatraemia in 26 (79%) hyperphosphataemia in 20 (69%) and hypokalaemia in 19 (63%) calves.

**Table 2:** Haematological and blood biochemical findings in calves with type-4 abomasal ulcer

Variable (mean ± sd or median, 25–75th percentiles)	Finding	n	Percent
Haematocrit (%) (n = 37; mean ± sd, 38.3 ± 10.9%)	Normal (30–35)	8	22
	Decreased (9–29)	7	19
	Increased (36–70)	22	59
Total protein concentration (n = 37; mean ± sd, 56.7 ± 11.6 g/L)	Normal (60–80)	13	35
	Decreased (32–59)	23	62
	Increased (81–89)	1	3
Fibrinogen concentration (n = 33; median, 6.0 g/L, percentiles 4–8 g/L)	Normal (4–7)	21	64
	Decreased (2–3)	2	6
	Increased (8–11)	10	30
Urea concentration (n = 34; median, 10.1 mmol/L, percentiles 7–16 mmol/L)	Normal (2.4–6.5)	7	21
	Increased (6.6–50.2)	27	79
Bilirubin concentration (n = 31; median, 4.2 µmol/L, percentiles 2.5–9.4 µmol//L)	Normal (≤ 6.5)	21	68
	Increased (6.6–40.1)	10	32
Sodium (n = 33; mean ± sd, 141 ± 5.3 mmol/L)	Normal (145–155)	7	21
	Decreased (126–144)	26	79
Potassium (n = 34; mean ± sd, 5.1 ± 1.7 mmol/L)	Normal (4–5)	9	27
	Decreased (2.6–3.9)	10	29
	Increased (5.1–9.0)	15	44
Chloride (n = 35; median, 98 mmol/L, percentiles 90–102 mmol/L)	Normal (96–105)	17	49
	Decreased 71–95)	14	40
	Increased (106–112)	4	11
Calcium (n = 30; median, 2.25 mmol/L, percentiles 2.1–2.4 mmol/L)	Normal (2.3–2.6)	8	27
	Decreased (1.7–2.2)	19	63
	Increased (2.7–4.4)	3	10
Magnesium (n = 30; mean ± sd, 0.97 ± 0.27 mmol/L)	Normal (0.8–1.0)	11	37
	Decreased (0.3–0.7)	7	23
	Increased (1.1–1.7)	12	40
Inorg. phosphorus (n = 29; median, 3.26 mmol/L, percentiles 2.3–4.1 mmol/L)	Normal (1.3–2.4)	9	31
	Increased (2.5–9.2)	20	69

Twenty-three (67%) calves had leukocytosis (Table 3), and 20 of 23 (87%) calves had increased numbers of segmented neutrophils and 18 (78%) had increased numbers of band neutrophils (left shift). Regenerative left shift was detected in 14 (61%) calves and degenerative left shift in 4 (17%). Eosinopenia was seen in 20 (87%), monocytosis in 11 (48%) and lymphocytosis in 8 (35%) calves.

**Table 3:** White blood and differential blood count in calves with type-4 abomasal ulcer

Variable (median, 25–75th percentiles)	Finding	n	%
White blood cell count (/µL) (n = 34; median, 13,400/µL, percentiles 7875–18,500/µL)	Normal (5000–10,000)	8	24
	Decreased (2300–4999)	3	9
	Increased (10,001–109,800)	23	67
Segmented neutrophils (/µL) (n = 23; median, 9250/µL, percentiles 5076—14,800/µL)	Normal (1230–3350)	1	4
	Decreased (120–1229)	2	9
	Increased (3351–83,440)	20	87
Band neutrophils (/µL) (n = 23; median, 3347/µL, percentiles 982—7245/µL)	Normal (0–300)	5	22
	Increased (301–19,060)	18	78
Eosinophils (/µl) (n = 23; median, 0/µL)	Normal (50–1000)	3	13
	Decreased (0–49)	20	87
Monocytes (/µL) (n = 23; median, 790/µL, percentiles 398–1132/µL)	Normal (100–900)	11	48
	Decreased (0–99)	1	4
	Increased (901–6040)	11	48
Lymphocytes (/µL) (n = 23; median, 3890/µL, percentiles 2848–5620/µL)	Normal (2190–5120)	12	52
	Decreased (980–2189)	3	13
	Increased (5121–16,280)	8	35

Venous blood gas analysis showed acidosis in 26 (84%) and decreased base excess in 15 (48%) calves (Table 4).

**Table 4:** Venous blood gas analysis in calves with type-4 abomasal ulcer

Variable (mean ± sd)	Finding	n	%
pH (n = 31; 7.31 ± 0.12)	Normal (7.41–7.45)	1	3
	Decreased (6.97–7.40)	26	84
	Increased (7.46–7.52)	4	13
pCO_2_ (n = 31; 48.9 ± 8.5 mmHg)	Normal (35–45)	7	23
	Decreased (34.7)	2	6
	Increased (46–72)	22	71
Bicarbonate (n = 25; 24.2 ± 9.3 mmol/L)	Normal (20–30)	10	40
	Decreased (9–19)	8	32
	Increased (31–50)	7	28
Base excess (n = 31; -0.4 ± 10.8 mmol/L)	Normal (− 2.0 to + 2.0)	7	23
	Decreased (− 21.0 to − 2.1)	15	48
	Increased (2.1–27.0)	9	29

### Abdominal ultrasonography and abdominocentesis

**Fig. 3:**
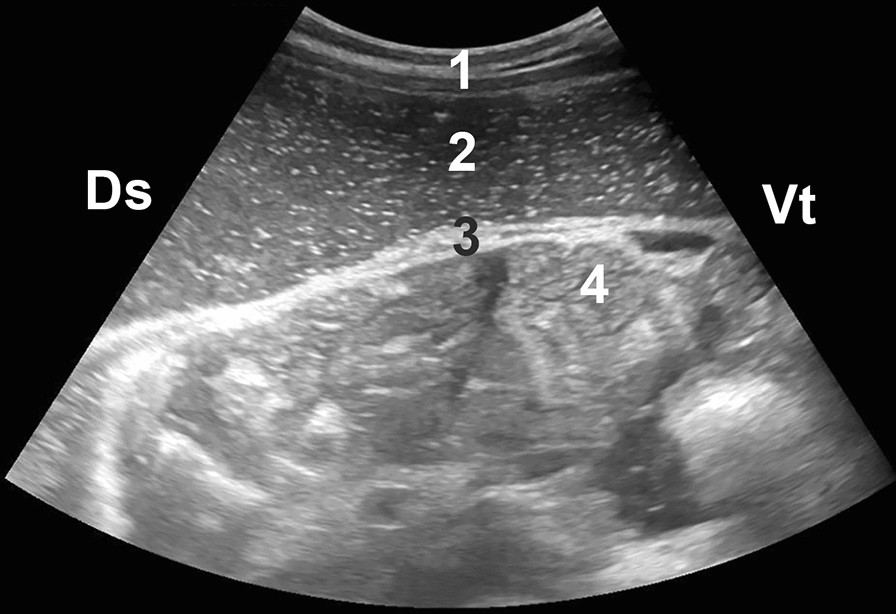
Ultrasonogram in a calf with diffuse peritonitis. Abdominal ultrasonogram of a three-month-old calf with diffuse peritonitis as a result of type-4 abomasal ulcer. The image was obtained from the right flank using a 5-MHz convex transducer. The abdomen contains a massive amount of fluid with gas inclusions (echogenic stippling). The small intestines are contained within the omental bursa. 1 Abdominal wall, 2 Abdominal fluid, 3 Greater omentum, 4 Small intestines, Ds Dorsal, Vt Ventral

**Fig. 4:**
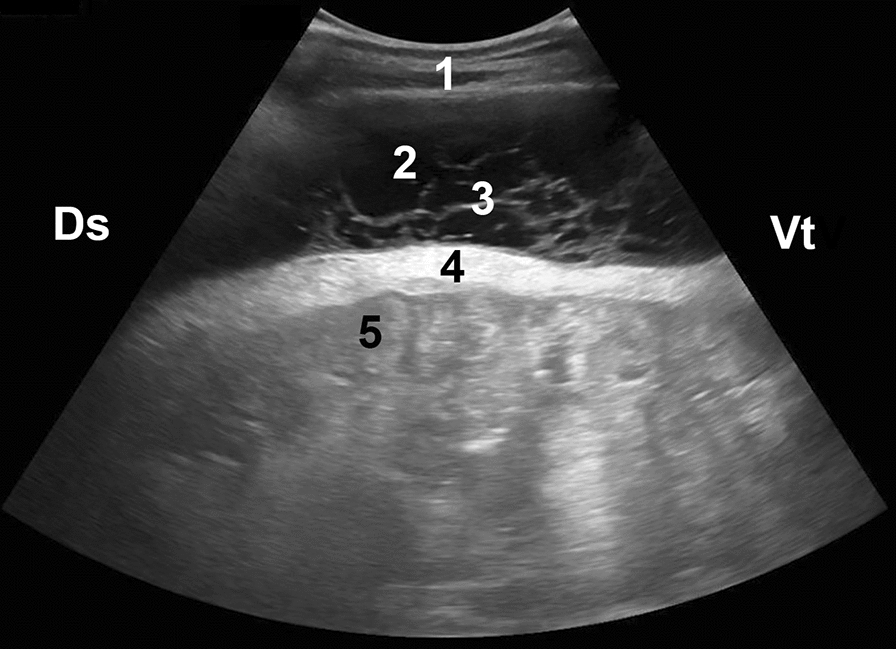
Ultrasonogram in a calf with diffuse peritonitis. Abdominal ultrasonogram of a 5-month-old calf with diffuse peritonitis as a result of a perforated abomasal ulcer. The image was obtained from the right flank using a 5-MHz convex transducer. The abdomen contains a large amount of fluid and fibrin septa. The small intestines are contained within the omental bursa. 1 Abdominal wall, 2 Abdominal fluid, 3 Fibrin strands, 4 Thickened greater omentum, 5 Small intestines, Ds Dorsal, Vt Ventral

The most frequent abdominal ultrasonographic findings were intestinal atony (68%), fluid (67%) (Fig. 3) and fibrin deposits or fibrin strands (58%) (Fig. 4) (Table 5). The abomasum was dilated in 13 of 36 (36%) calves and displaced to the left in two (6%). Thirteen (41%) calves had dilated small intestines, and four (11%) had gas inclusions in the abdomen. Of the 15 peritoneal fluid samples, 10 were turbid, 12 had brownish red, white or green discolouration and 12 were malodourous or had a sour smell.

**Table 5:** Ultrasonographic findings in calves with type-4 abomasal ulcer

Variable	Finding	n	%
Abomasum (n = 36)	Normal size	21	58
	Dilated	13	36
	Left displacement of the abomasum	2	6
Small intestine (n = 32)	Normal	12	37
	Dilated	13	41
	Empty	7	22
Small intestine, motility (n = 28)	Normal	9	32
	Absent	19	68
Free fluid in the abdominal cavity (n = 36)	Normal (no free fluid)	12	33
	Free fluid, anechoic (n = 7) or echoic (n = 17)	24	67
Further findings (n = 36)	Normal (no further findings)	11	31
	Fibrin, deposits (n = 16), strands (n = 5)	21	58
	Gas in the abdominal cavity	4	11

### Pathological findings

**Fig. 5:**
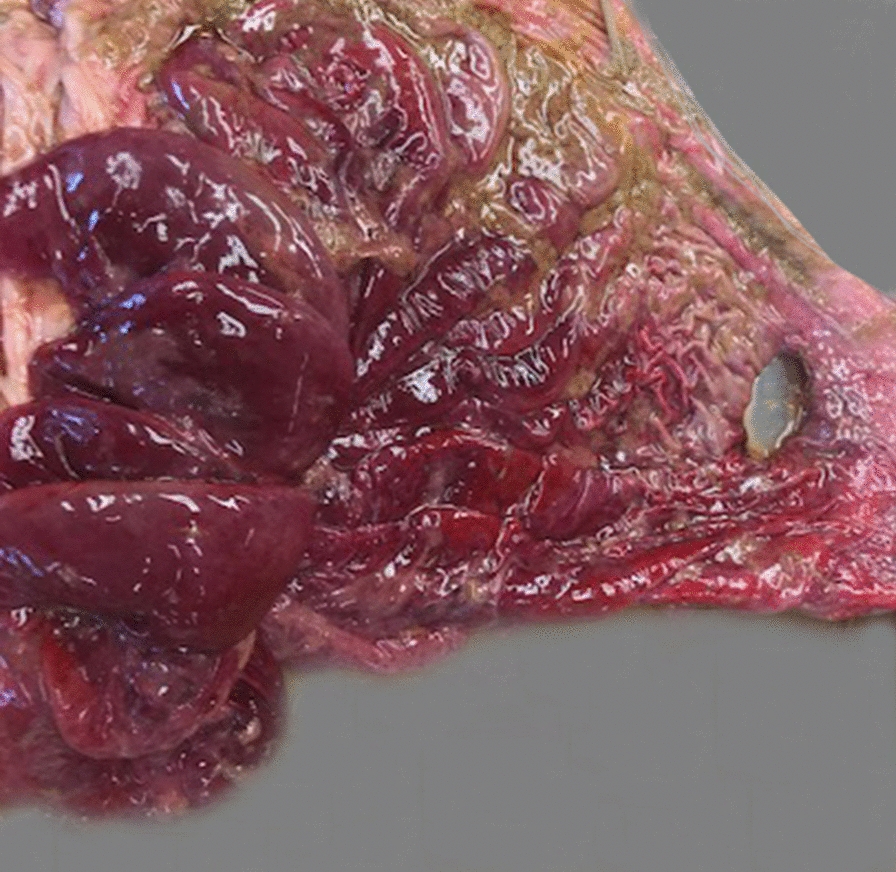
Postmortem findings in a calf with type-4 abomasal ulcer. Type-4 abomasal ulcer in a 6-week-old Fleckvieh bull calf. There is severe erythema and oedema of the abomasal mucosa

All calves had at least one perforated abomasal ulcer (Fig. 5) and diffuse peritonitis; 3 calves had two perforated ulcers. 23 (61%) calves had one, five (13%) had two, three (8%) had three and seven (18%) had more than three additional non-perforated (type-1) ulcers (Table 6). Of 37 calves, the diameter of the perforated ulcers ranged from 0.6 to 1.5 cm in six (16%); from 1.6 to 2.5 cm in nine (24%); from 2.6 to 3.5 cm in 10 (27%) and was more than 3.5 cm in 12 (32%). The abomasal mucosa was haemorrhagic in 13 (34%) calves and hypertrophied and oedematous in six (16%) calves each. Three calves had left displaced abomasum, and one calf had abomasal volvulus. The abomasal content was milky and had a green tinge in 34 calves and haemorrhagic in three, and one calf had a trichobezoar. In three calves the rumen contained milk, and in two a trichobezoar. Five calves had bronchopneumonia, two had lice and one had omphalitis.

**Table 6:** Postmortem findings in 38 calves with type-4 abomasal ulcer

Variable	Finding	n	%
Number of abomasal ulcers	One ulcer	23	61
	Two ulcers	5	13
	Three ulcers	3	8
	> 3 ulcers	7	18
Diameter of the abomasal ulcers (cm)	0.6–1.5	6	16
	1.6–2.5	9	24
	2.6–3.5	10	26
	> 3.5	12	32
	Not determined	1	2
Abomasal mucosa^a^	Normal	18	47
	Erythema/haemorrhage	13	34
	Thickened due to inflammation	6	16
	Oedematous	6	16
Position of the abomasum	Normal	34	89
	Left displacement of the abomasum	3	8
	Abomasal volvulus	1	3
Abomasal contents	Milky with green tinge	34	89
	Haemorrhagic	3	8
	Bezoar	1	3

^a^The sum of the percentages exceeds 100 because 5 calves had more than one lesion

### Final diagnosis

In all calves, the principal diagnosis was diffuse peritonitis associated with U4. Seventeen (45%) calves had one (n  =  16) or two comorbidities (n  =  1), which included bronchopneumonia (n  =  5), abomasal displacement/volvulus (n  =  4), ruminal drinking syndrome (n  =  3), pediculosis (n  =  2), persistent bovine viral diarrhoea virus infection (n  =  1), omphalitis (n  =  1) and enteritis (n  =  1).

## Discussion

Diagnosis of a perforated abomasal ulcer in a calf less than a week old was striking but has been described before [33]. The pathogenesis is not clear but it is conceivable that the ulcer started in utero. A report in human medicine described a 1-day-old term baby with melaena and haematemesis born to a woman who had suffered severe psychosocial stress in the second trimester [34]. Abomasal ulceration in a neonatal calf was attributed to stress of the dam, which was recumbent before parturition and then required veterinary assistance because of dystocia [33]. A relevant history was not available in the present case, and thus prenatal stress could not be determined. Two thirds of the calves described in this report were less than 2 months of age, which was in agreement with another study, in which most of the beef calves with abomasal ulcers were younger than two months [35]. In another study, more than half of 118 affected calves were less than eight weeks old, a third were between eight and 12 weeks and only about 10% were older than 12 weeks [15]. Of the calves in the present study, close to 20% were older than 3 months.

Several risk factors thought to be associated with abomasal ulceration were also encountered in this study. Just over half of the calves had received an NSAID, and approximately 13% had received a corticosteroid; both types of drugs have been associated with abomasal ulceration [15, 26, 27]. The risk of ulceration increases when these drugs are overdosed, administered for a prolonged period or given in combination. Detailed accounts of risk factors for abomasal ulceration have been published [3, 36].

Typical clinical signs of perforated abomasal ulcers in calves include abnormal posture (arched back, tucked-up abdomen and lowered head carriage) and an enlarged and tense abdomen, but signs of pain are noticeably absent [29]. These authors reported that 78% of affected calves held their muzzle in water without drinking and that a wet muzzle and submandibular area were common clinical signs in the calves at admission. In ten calves with U4, recumbency, tachycardia, hypothermia and pale mucous membranes were the predominant clinical signs [37]. In our study, the main clinical signs were poor general health and ensuing rumen atony and abdominal guarding because of diffuse peritonitis. A tense abdominal wall, an arched back and tucked-up abdomen reflect parietal pain, which is typical of peritonitis. Signs of visceral pain, such as colic or the assumption of a “sawhorse” stance, were relatively rare and were seen in 13 and 8% of calves, respectively. Cows with U4 have a similar clinical presentation that included illness associated with poor appetite or anorexia (100%), abdominal guarding (81%), congested scleral vessels (77%), rumen atony (73%) and tachycardia (68%) [12]. The principal clinical signs of the calves in the present study were related to shock caused by diffuse peritonitis. In addition to poor general health, we noted dehydration (reduced skin elasticity in 95% and enophthalmus in 73% of the calves) and lowered skin surface temperature (66%) and tachycardia (66%); the latter two signs were thought to be caused by centralisation of circulation. Other authors reported that signs of shock are rare in calves with U4 [15].

The laboratory findings were in agreement with published data [25, 28, 37] and included haemoconcentration, hypoproteinaemia, hyperfibrinogenaemia, azotaemia, leukocytosis and hyponatraemia [25, 28, 37, 38]. The most frequent laboratory abnormalities of this study were increased numbers of segmented neutrophils (87%) and eosinopenia (87%); the former reflected severe inflammation, and the latter, together with leukocytosis, neutrophilia, lymphopenia and monocytosis were consistent with a stress leukogram [39, 40]. In 78% of calves the increased number of segmented neutrophils was accompanied by an increase in band neutrophils, which indicated that the bone marrow was functional and capable of meeting the increased demand for leukocytes caused by peritonitis. In many cases, cattle with severe inflammation are not able to meet the demand for leukocytes and therefore develop leukopenia, often associated with a degenerative left shift [41]. Degenerative left shift was seen in 44.5% of cows with toxic mastitis [42] but in only 17% of calves of the present study. Not surprisingly, 61% of the calves had a regenerative left shift compared with only 16.4% of cows with toxic mastitis. This suggests that leukocyte consumption exceeded bone marrow production in cows with toxaemia, whereas an equilibrium between production and consumption occurred in calves with U4. At the time of sampling, most calves were peracutely affected, whereas most of the cows had acute to subacute toxic mastitis. It is likely that the total leukocyte count of calves in a later stage of the disease would have reflected bone marrow exhaustion similar to cows with toxaemia. Another possible explanation for the leukopenia seen in cows with toxic mastitis is that experimental intramammary infection with *Escherichia coli* or *Klebsiella pneumoniae* was shown to cause a decrease in neutrophils within 16 h of infection [43, 44]. Other laboratory abnormalities, subsequent to shock-related centralisation of circulation, were increased haematocrit in 59% and azotaemia in 79% of the calves. Haemoconcentration is usually accompanied by increased total protein concentration but this was not so in cows with U4; 69% of cows had haemoconcentration but only 11% had increased total protein concentration [12], 29% had concomitant hypoproteinaemia and 20% had increased haematocrit and concurrent hypoproteinaemia [12]. Haemoconcentration accompanied by hypoproteinaemia was seen in only 32% of the calves. Haemoconcentration combined with normo- or hypoproteinaemia is associated with a loss of protein-rich fluid [45], and in cattle with clinical signs of peritonitis provides important diagnostic information reflecting the massive loss of fluid and protein into the abdominal cavity [46]. Fibrinogen was increased in only 30% of the calves. Fibrinogen is an acute-phase protein that can increase in blood within two to three days of the onset of acute inflammation [47]. However, at the time of sampling, the median duration of illness was only 1 day. In contrast, hyperfibrinogenaemia was seen in 45% of cows with U4 after a median duration of illness of 2 days [12], in 39% of cows with acute mastitis after a median duration of illness of 2 days [42] and in 69% of cows with traumatic reticuloperitonitis after a median duration of illness of 4 days [48]. Thus, fibrinogen concentration is not a reliable indicator of peritonitis caused by a U4 in calves because the clinical signs usually precede hyperfibrinogenaemia. In contrast to an earlier study [28], acidosis was a common finding and was seen in 84% of the calves compared with 49% of cows with type-4 ulcer [12]. Acidosis is attributed to dehydration and shock-related anaerobic metabolism [49]. However, the diagnostic utility of metabolic acidosis for practitioners is limited because point-of-care venous blood gas analysis is not usually available and acidosis also occurs in calves with diarrhoea or ruminal drinker syndrome.

The main ultrasonographic finding in calves with U4 was free fluid in the abdomen, which was seen in 24 of 36 calves. However, the ulcer itself could not be visualized in any of the calves. The ultrasonographic visualisation of a perforated abomasal ulcer in a cow was only recently reported [50]. Fibrin deposits and/or fibrin strands were seen in 21 calves reflecting the severity of peritonitis. Of 75 cows with perforated abomasal ulcers, 87% had ultrasonographic evidence of localised or diffuse peritonitis [12]. Even though these ultrasonographic findings are near pathognomonic for peritonitis, a sample of fluid should be aspirated and analysed [51]; the samples obtained in the present study without exception were characterised by inflammatory changes. Even though the perforated ulcers could not be directly visualised, the ultrasonographic findings facilitated diagnosis and were a prognostic aid. Diffuse fibrinous peritonitis almost always has a poor prognosis and therefore this finding aids in decision making; the only feasible option in such cases is euthanasia, and other measures such as exploratory laparotomy or medical treatment can be forgone. Ultrasonographic examination of the abdomen is straightforward and should always be carried out when abomasal ulcer is the tentative diagnosis. A recent experiment designed to investigate whether the measurement of sucrose in blood after oral administration in calves would allow an in-vivo diagnosis of abomasal ulcers—based on the hypothesis that affected calves have increased gastric permeability—showed no difference in sucrose concentration between calves with and without abomasal ulcers [52].

## Conclusions

Perforated abomasal ulcers are associated with severe illness in calves. Clinical examination and measurement of the haematocrit and plasma protein combined with abdominal ultrasonography allow a presumptive diagnosis in most cases. Exploratory laparotomy may be used to confirm the diagnosis.

## Data Availability

The datasets used and analysed for this study are available from the corresponding author on reasonable request.
